# “Reasons … the reasons that we’re here:” Young pediatric nephrologists reflect on the profession

**DOI:** 10.3389/fped.2022.963811

**Published:** 2022-10-26

**Authors:** Alexandra J. Wenig, Emily J. Stonebrook, O. N. Ray Bignall

**Affiliations:** ^1^ Department of Pediatrics, Nationwide Children's Hospital, Columbus, Oh, United States; ^2^ Department of Pediatrics, Division of Nephrology and Hypertension, The Ohio State University College of Medicine, Columbus, Oh, United States

**Keywords:** women in medicine (WIM), pediatric workforce, pediatric nephrology, young pediatrician, young nephrologists, career decisions

## Introduction

The pediatric nephrology workforce shortage is a crisis that continues to demand the full attention of our field. To meet this challenge, nephrologists must closely examine the current state of the field; changing attitudes and perspectives among trainee pediatricians; as well as challenges and opportunities inherent to the work of pediatric nephrology that impact the decision of trainees to pursue this career. Ashoor et al. recently critically examined the crisis, and called the field to action with a broad range of approaches: building broad-based academic coalitions to engage and develop future nephrologists; addressing reimbursement and practice policy issues; adjusting the training duration; and aggressively addressing burnout and professional wellness (1). Jhaveri et al. demonstrated that intentional activities such as utilizing innovative teaching methods and engaging residents in scholarship and mentorship are critical to increasing interest in nephrology (2). Additionally, pediatric kidney care is a field uniquely suited to child health professionals with an interest in social determinants of health; social and health care justice; medical education and mentorship; and research and innovation. Exposure to the breadth of opportunity for a career in nephrology is essential for inspiring future trainees.

There are a total of 1,124 board-certified pediatric nephrologists in the United States (3). There were 50 new pediatric nephrology board certifications in 2020, a number which has declined from the recent peak of 89 certifications in 2014 (3). This attrition stands in contrast to other pediatric subspecialties which saw much higher new certifications in 2020, including hematology/oncology (297), cardiology (294), gastroenterology (202), and endocrinology (142) (3). Outside pressures have been theorized to contribute to the challenges in recruiting pediatricians to subspecialize in nephrology, including perceptions of the complexity of the field, lack of fair renumeration, and inadequate didactics during time in training (1, 2).

As pediatricians in varying stages of our careers, we each have experienced unique factors which have attracted us to the field. Key features like mentoring relationships; prospects for a career in research and innovation; and opportunities to address health and social justice made a difference for us, and may make a difference for others. In this piece, we explore the “reasons… the reasons that we’re here: (4)” *the reasons to look* at a career in pediatric nephrology, *the reasons to join* this exciting field, and *the reasons to remain* and develop a fulfilling and meaningful career.

## Reasons to look

When it comes to considering a career in pediatric nephrology, there is more than meets the eye of most medical students. Many young trainees dismiss the possibility of becoming a nephrologist early on, often because of the perceived complexity of renal physiology and the lack of exposure to pediatric nephrology as compared to other pediatric specialties while in medical school. But there are so many reasons why, if introduced intentionally, nephrology can be particularly appealing to a medical student. The American Society of Nephrology (ASN) has put great effort into making these reasons more apparent with their *United 4 Kidney Health Campaign* which emphasizes (1) early intervention, (2) transforming kidney transplantation, (3) accelerating innovation, and (4) achieving kidney health equity (5). Continued work at both institutional and national levels to emphasize the exciting ways the field is advancing – including in the care of children with kidney disease – is key to attracting trainees to the field.

The importance of increasing the visibility of pediatric nephrologists early in the medical school experience cannot be overstated. The nephrology community is full of educators who are eager to teach, mentor, and develop learners, and to do so in a way that is tailored to the unique gifts and passions of trainees. Early mentorship can allow pediatric nephrologists to help medical students draw parallels between their own career interests and the opportunities that exist within nephrology (6, 7). Opportunities to connect trainees early on with the broader nephrology community, like the ASN Kidney STARS and the American Society of Pediatric Nephrology (ASPN) Travel Award programs, allow medical students and residents to see a consistent degree of investment in trainees before fellowship (8). These opportunities for more regional and national engagement have the added benefit of making evident the close-knit, enthusiastic, collaborative culture that exists amongst pediatric nephrology providers, which is one of the field's greatest strengths.

One of the most encouraging qualities of individuals entering medical training today is their commitment to addressing social injustice as healthcare providers. The social aspects of nephrology may not be thought of first by those considering a kidney health career, but once they are seen, they can be a powerful incentive for trainees who desire to match their love of medicine with their love of humanity. Indeed, the reality is that pediatric nephrologists are a group of doctors who care immensely about the social determinants of kidney health, and often seek to heal the social factors that place their patients at risk. Many pediatric nephrologists dedicate significant time to advocacy for their patients and kidney health at local, state, national, and international levels (9). There is much value in finding ways for trainees to connect with a career in pediatric nephrology by engaging them in kidney health advocacy; perhaps it will prove to be one of the biggest draws to the field for this generation of future pediatric nephrologists.

## Reasons to join

The pediatric nephrologist is afforded an exceptional opportunity to practice in a wide variety of settings, and thus is privy to diverse clinical experiences and collaborations. One can function as an intensivist managing kidney replacement therapy in the intensive care unit; a hospitalist managing acute kidney injury on the wards; and a primary care provider for a kidney transplant recipient! A career in this field can look different from year to year, from family to family, and from patient to patient. For nephrologists, the beauty of this variety is a career that can grow and evolve with time and as their own unique gifts and passions, both personally and professionally, develop.

There are many pathways available for individuals to align their career with scholarly interests. Academically, the diversity of patients and disease processes gives way to an expansive range of research opportunities. Clinically there is the ability to practice as a general nephrologist or to focus on one specific aspect such as dialysis, transplant, or glomerular disease. The breadth of scope within the field brings with it the flexibility for trainees and young physicians to more easily change directions within their careers (10). Rather than being forced to adhere to a rigid career path set out from before the start of fellowship training, the trainee is able to explore the spectrum of opportunities within the field which, in turn, gives way to more defined interests and career paths.

Synergistic with a wide variety of clinical and academic opportunities is the opportunity for mentorship from committed nephrologists and kidney health professionals who can help junior faculty and trainees discover their passions and develop their interests. Furthermore, the kidney community is full of people who will not only assist trainees in achieving professional goals, but also take a genuine interest in their personal goals. Trainees are increasingly prioritizing access to a more holistic type of mentorship that speaks to both their work and their life outside of work (11). This range in specializations and opportunities for evolution in career direction make nephrology an attractive career choice for those wishing to balance these factors and integrate their career with a fulfilling personal life. Dedicated mentors play a critical role in not only highlighting the thrills of nephrology, but also in helping to guide trainees across the threshold as they begin their own journey in this exciting subspecialty. In fact, for many nephrologists, the enthusiastic engagement and support of a committed mentor has been the reason to join the field.

## Reasons to remain

Among the most compelling reasons to remain in pediatric nephrology is the long-term satisfaction and sense of purpose afforded by the profession. The decision to stay is facilitated by the availability of numerous occupational avenues: in addition to clinical care, sponsored research, advocacy, medical education, and hospital leadership add richly to the career (12). This variety is often also accompanied by flexibility for both trainees and faculty who may have competing personal or familial demands. Depending on the practice setting, faculty can find potentially flexible work hours, part-time positions, and the ability to transition into new roles or practice settings throughout their career.

The job of a pediatric nephrologist is inherently collaborative. Due to the prevalence of conditions like acute kidney injury, electrolyte abnormalities, hypertension, and fluid imbalance, pediatric nephrologists find themselves caring for patients – and with colleagues – from across the hospital and outpatient clinics. As a result, nephrology is a highly cooperative specialty necessitating the interaction with a wide variety of health care professionals and trainees. Similarly, whether in conducting research, manuscript writing, clinical care, or legislative advocacy, team-based practice within pediatric nephrology is the norm and brings about lasting relationships with colleagues, both from around one's institution and around the world.

A wide spectrum of unique experiences – from stimulating research and clinical innovations, to meaningful contributions to education and advocacy – provide many compelling reasons for pediatric nephrologists to remain in this exciting discipline. What's more, the powerful, long-lasting relationships with children and adolescents living with kidney disease and their families imbue nephrologists with a tremendous sense of purpose in a role akin to the primary care physician. We experience the highest highs and the lowest lows with our patients. We may meet a family in the neonatal intensive care unit, follow the child through progression of chronic kidney disease, and have the privilege of making the call to the family when a kidney allograft becomes available, shepherding the whole family through the kidney transplant experience and beyond. To many, these longitudinal relationships with patients and their families are some of the most meaningful experiences in their careers, and keep humanism at the heart of an otherwise challenging job.

## So many reasons to share

The work of growing the field of pediatric nephrologists must be shared by each and every kidney health professional. The tremendous sense of satisfaction, comradery, and purpose shared by the vibrant and diverse community of pediatric nephrologists stands in contrast with the workforce challenges that we are facing across our field. Many students have been dissuaded from joining nephrology by antiquated notions about what it takes to be successful (“To be a nephrologist, you have to be the smartest doctor in the hospital…”), and have not been exposed to the incredible opportunities to practice a stimulating career marked by dedication, compassion, empathy, advocacy, and innovation. Let us commit ourselves to first and foremost sharing these reasons – the reasons that make this the most amazing subspecialty in all of pediatrics.

## Data Availability

The original contributions presented in the study are included in the article/Supplementary Material, further inquiries can be directed to the corresponding author/s.
